# Exploring undergraduate nursing students’ experiences towards home-based learning as pedagogy during the COVID-19 pandemic: a descriptive qualitative exploration

**DOI:** 10.1186/s12912-021-00788-9

**Published:** 2022-01-04

**Authors:** Yanan Hu, Jenna Qing Yun Ow Yong, Mui-Lee Cecilia Chng, Ziqiang Li, Yong-Shian Goh

**Affiliations:** 1 Alice Lee Centre for Nursing Studies, Yong Loo Lin School of Medicine, National University of Singapore, Clinical Research Centre (MD 11), 10 Medical Drive, Singapore, 117597 Singapore; 2 National University Hospital, National University Health System, Singapore, Singapore; 3 Institute of Mental Health, Singapore, Singapore

**Keywords:** Home-based learning, Pedagogy, Challenges, Nursing program

## Abstract

**Background:**

The global COVID-19 pandemic has led to the need for educators to explore online platforms in delivering lessons to students. Home-based learning is one of the most commonly-used teaching methods that allow learning to take place despite a physical separation between the students and the educators.

**Methods:**

A descriptive qualitative approach was used to explore the experiences of nursing undergraduates when using home-based learning as a pedagogy during the COVID-19 pandemic. Data were collected from twenty-three nursing students (*n* = 14 in year one; *n* = 9 in year two) of their full-time pre-registration nursing program in a public-funded university in Singapore. Semi-structured interviews using an interview guide was conducted through Zoom-based video-conferencing from November 2020 to January 2021. The interview lasted between 45 and 65 min (median = 45 min). Data collection took place concurrently with thematic analysis through Braun and Clarke’s six-step approach. This study was reported according to the Consolidated Criteria for Reporting Qualitative Research.

**Results:**

Three main themes identified during the data analysis were: (1) challenges of home-based learning, where students detailed their experiences and difficulties encountered during the process; (2) the effectiveness of home-based learning, which explored the pedagogy’s impact on the students’ learning experience; and (3) students’ motivation to learn, where the effects on student morale and motivation in partaking in learning tasks were discussed.

**Conclusions:**

Results from this study suggested that universities should incorporate more home-based learning opportunities as home-based learning to continue playing a crucial role in the foreseeable future. Universities should continue to incorporate more home-based learning opportunities into the existing nursing curriculaa in order to test their capacities and address technical challenges in online learning. Future studies should also consider incorporating other pedagogical strategies when conducting lessons online.

## Introduction

The promulgation of the 2019 coronavirus disease (COVID-19) as a global pandemic in March 2020 [1] has led many countries to instituting nationwide lock-downs and social distancing among their citizens [2]. While unavoidable, such public-health measures have presented unprecedented logistical constraints across many industries worldwide. In education, severe disruptions have been reported, wherein approximately 94% of the global student population (1.6 billion learners) has been affected, ranging from students in elementary schools to those in tertiary institutions from more than 190 countries [3, 4].

In nursing education, both theoretical lectures and practical skills-based learning are emphasised: nurses are professionally required to possess not only bedside skills such as managing medications, administering injections, and wound care [5], but also soft skills such as therapeutic communication, counselling, and behavioural management [5]. In this regard, pandemic-related restrictions in face-to-face tutor-and-student interactions and hospital-based patient-care opportunities have presented substantial barriers to nursing education [6]. Given the consequent lack of opportunities to hone these crucial skills, students have expressed concerns over potential disadvantages in their future careers as compared to other cohorts who have not undergone home-based learning (HBL) [7]. Accordingly, a pedagogical transition from physical learning to virtual education has been advocated for educational institutions worldwide [3, 4].

### Home-based learning

Despite its traditionally adjunctive role alongside face-to-face pedagogies [8], HBL or e-learning has garnered attention in the context of a global pandemic. HBL commonly refers to the incorporation of Information and Computer Technologies (ICT) into the learning process and may permit physical separation between the learner and the instructor [9]. It involves a combination of synchronous technology such as real-time video-conferencing and asynchronous technology such as emails or pre-recorded lectures [10] to deliver the necessary content to students. This allows students to enjoy not only off-site access to their educators and learning materials, but also virtual participation in discussions without the need for physical presence in school [11]. Nonetheless, despite the current generation of learners being “digital natives” [12], HBL still presents a plethora of challenges: technical issues, engagement problems, lack of practical application and guidance, difficulties in collaborating with others, and difficulties in balancing schoolwork with social life during HBL [13].

Despite copious literature on e-learning for nursing students over the past decades [14], only one study [15] has hitherto examined their qualitative experiences during the first month of the COVID-19 pandemic. A distinction is noteworthy: students in the pre-pandemic era could be subjected to blended-learning approaches (simultaneous face-to-face and online instruction) or one-time e-learning programs [14], whereas students in the midst of a pandemic would be required to complete entire modules online [15]. In this context, understanding the perceptions and expectations of HBL among these students is critical to the provision of the necessary pedagogical resources for professionally grooming them. Therefore, the current study aimed to explore nursing students’ experiences of using home-based learning as a pedagogy during the COVID-19 pandemic. The findings were envisioned to insightfully inform the future deployment of HBL and e-learning initiatives in nursing education.

## Methods

### Design

A qualitative descriptive approach was used for exploring of the experiences of home-based learning among nursing undergraduates in a public-funded university in Singapore that ran undergraduate to post-graduate nursing programmes. This study was reported according to the Consolidated Criteria for Reporting Qualitative Research (COREQ), a 32-item checklist (Tong et al., 2007).

### Participants

Participants were recruited from the pre-registration nursing programme through convenience sampling. They were informed via an announcement made in their first synchronised online lectures, followed by an email invitation circulated by the departmental administrative support team. The undergraduates were assured that their participation/non-participation in this study would not impact their results and that this research did not form a part of their modules.

### Setting

Singapore reported its first case of COVID-19 infection in late January 2020 [16]. By February 2020, the situation had deteriorated, prompting the Singapore government to escalate its Disease Outbreak Response System Condition (DORSCON) to the ‘orange’ alert (i.e. moderate severity and spread of disease). A two-month ‘circuit breaker’ period from April to June 2020 followed, alongside safe-distancing measures and by-default work-from-home measures for all non-essential services [17]. Accordingly, all institutes of higher learning (universities) in Singapore implemented home-based learning which lasted two semesters within the Academic Year from August 2020 to July 2021. This research was conducted in an autonomous University where a range of nursing programs that includes pre-registration nursing programs to post-graduate programs were offered. The pre-registration nursing course is a three-year Bachelor of Science (Nursing) program with an option for students to continue with a fourth year if they wished to pursue their Honours year.

### Data collection

Data were collected from November 2020 to February 2021 through individual semi-structured interviews after the Semester 1 of home-based learning was over. The participants completed a short socio-demographic questionnaire which included their age, gender, year of study and previous educational background before their interviews. Based on a review of the literature (Hasan and Bao, 2020; Moule et al., 2010), semi-structured questions were developed by the research team such as, “what is your understanding of ‘home-based learning?” and “what are the challenges you faced when trying to learn nursing topics at home?”. The interviews were conducted through video-conferencing on Zoom (an online meeting platform) by a research team member who had no direct grading responsibilities in the nursing program. Conducted in English, the interviews lasted 45 to 65 min (median of 45 min) and were recorded with the participants’ consent. Data saturation was used to determine the final sample size required in this study (Hennink et al., 2017).

### Data analysis

The content of the interviews was manually transcribed verbatim immediately after each session to ensure accuracy and minimise distortion of meanings and interpretation of data (Blome and Augustin, 2015) while maintaining a constant awareness of the researchers personal assumptions from the beginning to the end of the research process [18]. Thematic analysis using the inductive approach was then conducted independently by two researchers (YH and YSG) in the team through the six-step process suggested by Braun and Clarke (2006) to identify, analyze, organise, describe, and finalise the themes generated from the data. Initial codes were first generated from the dataset and then collated for the identification of the themes. The researchers then fitted the coded extracts and generated a concept map to review and named the themes. The data were then reviewed by the research team to ensure the authenticity and accuracy of findings and meanings. The codes were rrearranged if when there were any new additional of new codes or during the removal of existing codes to enhance the coherence between the codes and their respective themes. Finally, the research team selected the most appropriate verbatim to support the identified themes allowing the readers to comprehend its important aspects.

### Rigor of findings

Methodological rigor and trustworthiness were established based on the criteria set by Lincoln and Guba (1985), by which credibility, transferability, and dependability were appraised. Firstly, the principal investigator performed post-transcription member-checking for credibility and interpretative accuracy (Saunders et al., 2018). This was done by returning transcribed and interpreted data to the participants for verification of validity. Secondly, the availability of the context of the study and its participants allowed readers to evaluate the transferability of findings (Saunders et al., 2018). Thirdly, an audit trail was produced, detailing the research process for dependability and potential replication of findings (Saunders et al., 2018). Lastly, a reflexive journal detailing thoughts, feelings, and reactions was kept to maintain objectivity and minimise the influence of bias on the results (Cope, 2014).

### Ethical considerations

Ethical approval was obtained from the Institutional Review Board of National University of Singapore (NUS IRB-2020-132). The study was assessed as posing no more than minimal risk and therefore granted the use verbal consent with exemption of written consent as it involved the participants attending a one-hour interview session. The participants were informed of the purpose of the study and that they can decline to answer any questions which they may feel uncomfortable during the interview session during recruitment. They were also assured of strict anonymity with the assignment of numerical representations for de-identification during transcription.

## Results

### Demographic characteristics

This study involved 23 participants with a mean age of 25.04, of whom 17 were females. All participants were from either Year One or Two in the nursing undergraduate program. (See Table 1).

**Table 1:** Demographic characteristics

Participant	Age	Gender	Year of Study within the Nursing program	Previous Educational Background	Interview Duration (Minutes)
1	27	Female	Year 2	Bachelor’s Degree	45
2	24	Female	Year 2	Bachelor’s Degree	45
3	48	Female	Year 2	Doctoral Degree	55
4	20	Female	Year 1	GCE ‘A’ Level	40
5	24	Male	Year 2	Diploma	40
6	30	Female	Year 2	Bachelor’s Degree	40
7	21	Female	Year 1	GCE ‘A’ Level	40
8	24	Male	Year 2	Diploma	40
9	35	Male	Year 2	Bachelor’s Degree	45
10	20	Female	Year 1	GCE ‘A’ Level	45
11	21	Female	Year 1	GCE ‘A’ Level	50
12	43	Male	Year 2	Diploma	65
13	21	Female	Year 1	GCE ‘A’ Level	45
14	20	Female	Year 1	GCE ‘A’ Level	50
15	25	Male	Year 2	GCE ‘A’ Level	40
16	21	Female	Year 1	GCE ‘A’ Level	45
17	21	Female	Year 1	GCE ‘A’ Level	45
18	21	Female	Year 1	GCE ‘A’ Level	40
19	25	Female	Year 1	Diploma	45
20	21	Female	Year 1	Diploma	40
21	20	Female	Year 1	GCE ‘A’ Level	40
22	22	Female	Year 1	Diploma	40
23	22	Male	Year 1	GCE ‘A’ Level	45

#### Thematic findings

Three main themes were identified during the data analysis, which described the learning experiences of nursing undergraduates from Alice Lee Centre for Nursing Studies (ACLNS), Yong Loo Lin School of Medicine, National University of Singapore (NUS) during the COVID-19 pandemic: (1) challenges of home-based learning; (2) effectiveness of home-based learning; and (3) students’ motivation to learn.

### Theme 1: challenges of home-based learning

Online learning has been a pedagogy familiar to the nursing undergraduates, given the existing inclusion of an e-learning week in every semester in their undergraduate program. However, the learning curve for many students steepened during the COVID-19 pandemic, upon the introduction of home-based learning for all lessons by the university, in response to the governmental safe-distancing measures. Many participants reported difficulties in handling technological challenges (such as poor internet connection, outdated software, and denied access to online activities) that followed such home-based learning. These issues, when unresolved in a timely manner, caused distress such as irritability or anxiety among them.

*“I just couldn’t get in [the Zoom class]; I have not started everyone already in class. I got panic; I don’t know what I can do... it makes me feel like crying” (P2).*

The struggle with such challenges was further amplified during the examination period where all face-to-face examinations were conducted on an online platform such as Examsoft (Examsoft, 2021). Many participants reported that this was an exhausting and anxiety-provoking experience.

“*We couldn’t get into teams...it gave us a lot of stress with heart-pumping rapidly not knowing what could be done next...” (P10).*

Even participants who themselves had not experienced problems with accessibility reported unpleasant learning experiences: one of them recounted the following.

“*along the way, if someone got into technical issues (laughs) … I’ll be wasting my time scrolling up and down until all of us can start our lessons together” (P12).*

Apart from the technical problems, some participants expressed difficulty in paying attention during their lessons. Many attributed this to the lack of a conducive learning environment in a domiciliary setting, aggravated by having the presence of their household during the circuit-breaker period from April to June 2020.

*“...during CB (circuit breaker), my entire family is at home. So like, it is a bit noisy. It was also very hard to find a quiet spot because like, I share the room with my siblings.” (P16).*

Even for the participants who had their study room, there were still other forms of distractions such as pets, household chores, and entertainment (such as televisions and mobile phones).

*“it’s rather challenging for me, so people at home might think that you are quite free... it is very hard to establish a separation between schoolwork and home duties” (P15).*

Finally, with the implementation of home-based learning, the participants had spent substantially more time viewing digital screens for their online lessons. Many participants thus reported experiencing “Zoom fatigue” such as difficulty in concentrating, eye strain, or lethargy, thereby advocating breaks in between long online teaching sessions. Therefore, participants suggested that breaks should be made available in between long online teaching sessions.

*“I feel tired when I look at the screen for too long, my eyes also very tired... I can only focus on first two hours... for the third and fourth, gone already, I can’t even concentrate...” (P6).*

### Theme 2: the effectiveness of home-based learning

Despite the challenges faced by the participants during home-based learning, the majority positively regarded their learning experiences, particularly with the use of pre-recorded online lectures. They reported that such recordings provided them the convenience and flexibility to review lectures at their preferred time, location, and pace without worrying that they might miss the content.

*“I liked pre-recorded lectures... because we get to replay them; replaying is very important for me because I’m very bad at picking up whatever the lecturers said in the first instance” (P8).*

Additionally, the participants could also repeatedly access the lectures to enhance their understanding.

*“I think having pre-recorded lectures is good for a slow learner like me, I can listen to the lecture again... which helped me... I can see my grades improved for that module when I listened two to three times” (P19).*

Lessons on nursing skills that had previously been conducted face-to-face were converted to pre-recorded videos prepared by the faculty demonstrating step-by-step skills. Despite the value of such these videos, the lack of face-to-face practical sessions predisposed many participants to feel incompetent and unconfident to practise them on patients when they would eventually be allowed to restart their clinical practice.

*“...with the COVID-19 situation, our practical sessions are now being taught using pre-recorded videos, and we do not have contact time to practice with our tutors...because of the lack of practice, I am worried next time we go for our clinical attachment...we don’t know how to do.” (P5).*

Many participants reported that, among the online learning platforms, Zoom tutorials were the best alternative for face-to-face tutorials. They remarked that the tutors were able to engage their students during online classes by posting questions, holding smaller sub-group discussions with breakout-room features, or using edu-gaming platforms such as Kahoot to capture individual attention. Moreover, several participants found that online tutorials were effective in encouraging self-directed learning.

*“...I felt the need to do my part of work more efficiently for online tutorials compare with physicality tutorials...It’s like, now I prepare more before attending the tutorial.” (P7).*

Another common challenge shared among the participants was the elevated load of reading materials provided during home-based learning compared to face-to-face learning sessions. Thus, some admitted that they had prioritised selected readings that they deemed would be more useful for examinations.

*“I cannot finish all the materials before the exam. Although I know that the additional reading will help us... I don’t have time to go for additional reading” (P2).*

### Theme 3: students’ motivation to learn

Despite the logistical convenience and flexibility of home-based learning, some participants reported their proclivity to procrastinate in their learning since they were in the comfort of their home.

*“...staying at home doesn’t help, being a student, it just breeds complacency, I felt that I have more time and there is no rush to study” (P21).*

Therefore, self-discipline became more crucial for these participants. As explained by Participant 6, there was a need to be more disciplined to complete learning tasks effectively through producing one’s own daily or weekly timetable.

*“I fixed the schedule for myself. So today’s right, I will list down these are all the stuff I need to do.” (P6).*

Additionally, several participants emphasised the importance of interactions between students: the virtual pedagogy affected their academic performance since learning took place not only by class attendance, but also through peer-to-peer interactions.

*“I’m just so worried that I cannot learn as much as what I see now from my classmates are only their social media post which serves no bearings in terms of my learning journey.” (P14).*

Accordingly, some participants strategised to arrange small study groups via online platforms or (where permissible) return to the campus to study together, thus reporting improved motivation to undertake home-based learning.

*“...the simple things like even like seeing your friends... sitting in side by side with your friends, it boosts your mood and motivation to study. Yeah, the environment plays a huge role in cultivating this learning culture in every student.” (P15).*

## Discussion

The COVID-19 pandemic has led to school closures and the conversion of all face-to-face lessons to home-based learning at all institutes of higher learning in Singapore. Thus, home-based learning has become a new norm within the higher education system in Singapore. This study explored the qualitative experiences of a cohort of nursing students in a public-funded university. The results obtained from the study showed that nursing students have been affected by the implementation of home-based learning in multifarious ways.

Obstacles to home-based learning such as poor internet connection and inaccessibility to online learning activities were encountered. Such technical challenges have been reported to both affect learning opportunities and cause psychological distress among learners [13, 19, 20]. While technical issues are inevitable in home-based learning, tutors may set aside time before classes to provide students the opportunity to troubleshoot technical difficulties without encroaching into the allocated lesson time. Teaching assistants or institutional technical support employees may also assist with these issues to alleviate excessive burden on tutors.

Moreover, lethargy and eye strain due to prolonged exposure to computers could further disrupt their concentration [21]. Therefore, occasional short breaks might be useful in alleviating these problems [22]. These breaks may be intentionally incorporated into classes by implementing additional intermittent time-out during lessons and ensuring that virtual classes are adequately spaced out to allow students time away from the screen. Additionally, it has been recommended that the duration of online classes should not exceed four hours daily to reduce ocular issues in students [23], which may in turn affect their school-related performance as observed in this study. Needless to say, students also have the responsibility to limit their personal screen time and take regular breaks to prevent fatigue from prolonged screen exposure.

Safe-distancing measures during the pandemic have brought a standstill to academic activities and raised unprecedented challenges to nursing students. Despite the challenges, most participants positively regarded online learning, especially the use of pre-recorded online lectures. The availability of pre-recorded lectures allowed flexibility and convenience with their learning as they could access and study at their preferred time, location, and pace [24].

Classroom teaching of nursing skills has always been a cornerstone for nursing programs. Pre-recorded videos have been used to complement traditional classroom teaching for years, for which positive results in knowledge and skills retention have been reported [25]. However, because of safe-distancing measures, the traditional classroom approach had to be replaced completely by the use of pre-recorded videos that demonstrated skills. The lack of classroom-based learning in this regard led to concern among the participants on their consequent inadequate preparation for clinical practice [26, 27]. Therefore, nursing faculties can in future consider exploring different pedagogy such as the use of virtual environments where nursing students can experience various scenarios and tasks as they might do in real life [28] allowing them to go through experiential learning beyond conventional learning methods [29].

The findings from this study also showed that the majority of the participants positively regarded online tutorials [30]. Many commented that having a well-designed online tutorial was similarly engaging and that they would often use the chat function available on the online meeting platform to clarify their doubts. The only drawback reported by some participants was the lack of non-verbal cues (such as eye contact), which in turn led to lower student-to-student or student-to-tutor interactions [31]. The decrease of social interaction in class might eventually lead to a decrease in cross-fertilization of ideas both among students and between students and tutors [32].

The flexibility of home-based learning gave the participants the autonomy to plan their studies [33], though being in the comfort of their home could also be counter-productive. Hence, a student’s intrinsic motivation to home-based learning would be a key element for their academic achievement [27, 34]. Most participants in this study took the initiative to be responsible for their learning by following their self-planned schedules or studying in small groups. The social interaction among peers served as motivational factors for students to study [35]. As aforementioned, students in this study were very receptive in engaging the chat function during online tutorials. Likewise, tutors may set up other communication platforms such as forums and informal group chats to facilitate the exchange of course-related knowledge and ideas among students as well as providing a space for peer interaction. These platforms may also encourage a sense of community and peer motivation for students who have difficulties adhering to learning tasks independently.

Furthermore, a caveat to the convenience afforded by home-based learning was the lack of a conducive environment at home for online learning classes due to disruptions by other family members, pets, entertainment, and even household chores [36]. Hence, the availability of a meaningful learning environment, the emotional climate, and peer interactions in small groups of students could boost their academic outcomes [15, 37].

### Strengths and limitations

In this study, the use of qualitative research techniques offered comprehensive insights into the experiences of nursing students using home-based learning during the COVID-19 pandemic. Despite geographical limitations which potentially restricted transferability of the results, the valuable experiences shared by the participants could be utilised by educators when developing home-based learning. Finally, the use of an online interviewing platform with video-conferencing features during data collection could have limited the researcher’s ability to discern non-verbal cues demonstrated by the participants.

## Conclusions

This study explored the experiences of nursing undergraduates after attending lessons for one semester through home-based learning platforms. Home-based learning is anticipated to continue playing a crucial role both during the pandemic and in the foreseeable future after it. The results from this study showed that universities should incorporate more home-based learning opportunities, since this would allow both students and universities to test their capacities and address technical challenges in online learning. Future studies should also consider other pedagogical strategies when conducting lessons on clinical skills.

### Implications to practice

The valuable insights provided by the students in this study will serve to inform future deployment of HBL and e-learning initiatives in nursing education. The transition from physical to virtual classes had both positive and negative effects on student learning. While students benefitted from pre-recorded lectures that accommodated each learner’s pace and allowed students the flexibility to plan their own schedules, many struggled with technical, environmental, physical, and social issues during HBL. Furthermore, students expressed concerns over the lack of development of clinical skills that are essential in the nursing field. Thus, it is crucial for teaching institutions to rise to the occasion to provide comprehensive support to students and address their concerns. Firstly, the use of virtual simulations may be further explored such that learning of clinical skills may take place in the comfort of the students’ homes. The field of virtual reality and simulations have been advancing in recent years [38] and have been used in nursing curriculums [39]. With the rapid expansion of virtual technology, it would be wise for teaching institutions to leverage these to provide distanced learning opportunities for students. Secondly, HBL has resulted in students having to learn in isolation while dealing with various issues that may interfere with their learning. Educators should take these into consideration while planning their lessons to maximize learning. For instance, additional support may be provided for students struggling to grasp course content and classes may be structured to allow for sufficient ocular breaks. Finally, the flexibility and convenience afforded by pre-recorded lectures may be utilized even after the pandemic, given the students’ receptiveness to it.

## Data Availability

The datasets used and/or analysed during the current study are available from the corresponding author on reasonable request.

## References

[1] World Health Organisation. WHO Director-General’s opening remarks at the media briefing on COVID-19 - 11 March 2020. 2020. Retrieved from Geneva: https://www.who.int/director-general/speeches/detail/who-directorgeneral-s-opening-remarks-at-the-media-briefing-on-covid-19---11-march-2020.

[2] World Health Organisation. Coronavirus disease (COVID-19) advice for the public. 2020 Retrieved from: https://www.who.int/emergencies/diseases/novel-coronavirus-2019/advice-for-public.

[3] OECD. The potential of online learning for adults: Early lessons from the COVID-19 crisis. 2020. Retrieved from: https://www.oecd.org/coronavirus/policy-responses/the-potential-of-online-learning-for-adults-early-lessons-fromthe-covid-19-crisis-ee040002/.

[4] UN Sustainable Development Group. Policy Brief: Education during COVID-19 and beyond. 2020. Retrievedfrom: https://unsdg.un.org/sites/default/files/2020-08/sg_policy_brief_covid-19_and_education_august_2020.pdf.

[5] Graham JM, Waddell C, Pachkowski K, Friesen H (2020). Educating the educators: determining the uniqueness of psychiatric nursing practice to inform psychiatric nurse education. Issues Mental Health Nurs.

[6] Alsoufi A, Alsuyihili A, Msherghi A, Elhadi A, Atiyah H, Ashini A (2020). Impact of the COVID-19 pandemic on medical education: Medical students’ knowledge, attitudes, and practices regarding electronic learning. PloS One.

[7] Daniel SJ (2020). Education and the COVID-19 pandemic. Prospects..

[8] Moule P, Ward R, Lockyer L (2010). Nursing and healthcare students’ experiences and use of e-learning in higher education. J Adv Nurs.

[9] Rodrigues H, Almeida F, Figueiredo V, Lopes SL (2019). Tracking e-learning through published papers: a systematic review. Comput Educ.

[10] Finkelstein J (2006). Learning in real time: synchronous teaching and learning online.

[11] Guven Ozdemir N, Sonmez M (2020). The relationship between nursing students’ technology addiction levels and attitudes toward e-learning during the COVID-19 pandemic: a cross-sectional study. Perspect Psychiatr Care.

[12] Cowey AE, Potts HWW (2018). What can we learn from second generation digital natives? A qualitative study of undergraduates’ views of digital health at one London university. Digit Health.

[13] Dhawan S (2020). Online learning: a panacea in the time of COVID-19 crisis. J Educ Technol Syst.

[14] De Caro W, Marucci AR, Lancia L, Sansoni J, Biondi-Zoccai G (2016). Case study 2: E-learning in nursing education in academic fields. Umbrella reviews: evidence synthesis with overviews of reviews and meta-epidemiologic studies.

[15] Ramos-Morcillo AJ, Leal-Costa C, Moral-García JE, Ruzafa-Martínez M (2020). Experiences of nursing students during the abrupt change from face-to-face to e-learning education during the first month of confinement due to COVID-19 in Spain. Int J Environ Res Public Health.

[16] Ministry of Health. Confirmed imported case of novel coronavirus infection in Singapore; multiministry taskforce ramps up precautionary measures. [press release]. Retrieved from https://www.moh.gov.sg/newshighlights/details/confirmed-imported-case-of-novel-coronavirus-infection-in-singapore-multi-ministry-taskforceramps-up-precautionary-measures.

[17] Ministry of Health. Strong national push to stem spread of COVID-19 [press release]. 2020. Retrieved from: https://www.moh.gov.sg/news-highlights/details/strong-national-push-to-stem-spread-of-covid-19.

[18] Sargeant J (2012). Qualitative research part II: participants, analysis, and quality assurance. J Grad Med Educ.

[19] Adedoyin OB, Soykan E. Covid-19 pandemic and online learning: the challenges and opportunities. Interact Learn Environ. 2020:1–13. 10.1080/10494820.2020.1813180.

[20] Hasan N, Bao Y (2020). Impact of “e-learning crack-up” perception on psychological distress among college students during COVID-19 pandemic: a mediating role of “fear of academic year loss”. Child Youth Serv Rev.

[21] Gillett-Swan J (2017). The challenges of online learning: supporting and engaging the isolated learner. J Learn Des.

[22] Lyu B, Zeng C, Deng S, Liu S, Jiang M, Li N, Wei L, Yu Y, Chen Q (2019). Bamboo forest therapy contributes to the regulation of psychological responses. J For Res.

[23] Ganne P, Najeeb S, Chaitanya G, Sharma A, Krishnappa NC (2021). Digital eye strain epidemic amid COVID-19 pandemic – a cross-sectional survey. Ophthalmic Epidemiol.

[24] Panigrahi R, Srivastava PR, Sharma D (2018). Online learning: adoption, continuance, and learning outcome - a review of literature. Int J Inf Manag.

[25] Öztürk D, Dinç L (2014). Effect of web-based education on nursing students’ urinary catheterization knowledge and skills. Nurse Educ Today.

[26] Forbes H, Oprescu F, Downer T, Phillips N, Mctier L, Lord B (2016). Use of videos to support teaching and learning of clinical skills in nursing education: a review. Nurse Educ Today.

[27] Farsi Z, Sajadi SA, Afaghi E, Fournier A, Aliyari S, Ahmadi Y, Hazrati E (2021). Explaining the experiences of nursing administrators, educators, and students about education process in the COVID-19 pandemic: a qualitative study. BMC Nurs.

[28] Lee Y, Kim SK, Eom M-R (2020). Usability of mental illness simulation involving scenarios with patients with schizophrenia via immersive virtual reality: A mixed methods study. PloS One.

[29] Piot M-A, Dechartres A, Attoe C, Jollant F, Lemogne C, Burn C (2019). Simulation in psychiatry for medical doctors: a system review and meta-analysis. Med Educ.

[30] Bolliger DU, Supanakorn S (2011). Learning styles and student perceptions of the use of interactive online tutorials. Br J Educ Technol.

[31] Kear K, Chetwynd F, Williams J, Donelan H (2012). Web conferencing for synchronous online tutorials: perspectives of tutors using a new medium. Comput Educ.

[32] Sohmer R, Michaels S, O'Connor MC, Resnick L, Schwarz B, Dreyfus T, Hershkowitz R (2009). Guided construction of knowledge. Transformation of knowledge classrom interactions.

[33] Al-Balas M, Al-Balas HI, Jaber HM, Obeidat K, Al-Balas H, Aborajooh EA (2020). Distance learning in clinical medical education amid COVID-19 pandemic in Jordan: current situation, challenges, and perspectives. BMC Med Educ.

[34] Chen KC, Jang SJ (2010). Motivation in online learning: testing a model of self-determination theory. Comput Hum Behav.

[35] Raufelder D, Jagenow D, Drury K, Hoferichter F (2013). Social relationships and motivation in secondary school: four different motivation types. Learn Individ Differ.

[36] Xie X, Siau K, Nah FFH (2020). COVID-19 pandemic–online education in the new normal and the next normal. J Inf Technol Case Appl Res.

[37] Wayne S, Fortner S, Kitzes J, Timm C, Kalishman S (2013). Cause or effect? The relationship between student perception of the medical school learning environment and academic performance on USMLE step 1. Med Teach.

[38] McCarthy CJ, Uppot RN (2019). Advances in virtual and augmented reality—exploring the role in health-care education. J Radiol Nurs.

[39] Goh YS, Owyong JQY, Seetoh YTM, Hu Y, Chng ML, Li Z (2021). Exploring pedagogies used in undergraduate mental health nursing curriculum: an integrative literature review. Int J Ment Health Nurs.

